# Establishing *Caenorhabditis elegans* as a model for *Mycobacterium avium* subspecies *hominissuis* infection and intestinal colonization

**DOI:** 10.1242/bio.012260

**Published:** 2015-09-24

**Authors:** Jamie L. Everman, Navid R. Ziaie, Jessica Bechler, Luiz E. Bermudez

**Affiliations:** 1 Department of Microbiology, College of Science, Oregon State University, Corvallis, OR 97331, USA; 2 Department of Biomedical Sciences, College of Veterinary Medicine, Oregon State University, Corvallis, OR 97331, USA

**Keywords:** *C. elegans*, Mycobacterium avium, Colonization, Host, Intestines

## Abstract

The nematode *Caenorhabditis elegans* has become a model system for studying the disease interaction between pathogens and the host. To determine whether the transparent nematode could serve as a useful model for *Mycobacterium avium* subspecies *hominissuis* (MAH) infection of the intestinal tract, worms were fed MAH and assayed for the effects of the bacterial infection on the worm. It was observed during feeding that viable MAH increases in the intestinal lumen in a time dependent manner. Ingestion of MAH was deemed non-toxic to worms as MAH-fed populations have similar survival curves to those fed *E. coli* strain OP50. Pulse-chase analysis using *E. coli* strain OP50 revealed that MAH colonize the intestinal tract, as viable MAH remain within the intestine after the assay. Visualization of intestinal MAH using histology and transmission electron microscopy demonstrates that MAH localizes to the intestinal lumen, as well as establishes direct contact with intestinal epithelium. Bacterial colonization appears to have a detrimental effect on the microvilli of the intestinal epithelial cells. The MAH ΔGPL/4B2 strain with a mutation in glycopeptidolipid production is deficient in binding to human epithelial cells (HEp-2), as well as deficient in its ability to bind to and colonize the intestinal tract of *C. elegans* as efficiently as wild-type MAH. These data indicate the *C. elegans* may serve as a useful model system for MAH pathogenesis and in determining the mechanisms used by MAH during infection and colonization of the intestinal epithelium.

## INTRODUCTION

*Caenorhabditis elegans* is a ubiquitous nematode which lives in soil and feeds on bacteria. Due to its transparent nature, simple and streamlined body structure, and its defined genome, *C. elegans* has become a widely used model organism for studying genetics, immunology, and host-pathogen interactions. The intestinal epithelium of *C. elegans* and human intestinal epithelial cells are quite similar, sharing comparable morphology, structure, and function, which includes acting as a first defense against invading pathogenic bacteria. The nematode has been characterized extensively as a model for bacterial pathogenesis of medically relevant organisms, including *Pseudomonas aeruginosa*, *Staphylococcus aureus,* and *Enterococcus faecalis* (Irazoqui et al., 2010; Garsin et al., 2001). It has been demonstrated that *C. elegans* can feed on fast-growing strains of mycobacteria including *Mycobacterium fortuitum* and *Mycobacterium marinum* (Couillault and Ewbank, 2002); however, the growth and feeding of *C. elegans* on slow-growing mycobacterial species has not been described. The ease of use of the nematode and the array of tools available make it a desirable candidate as a model system of other human pathogens of interest.

*Mycobacterium avium* subspecies *hominissuis* (MAH) is a member of the *Mycobacterium avium* complex and is an environmental bacterium known to cause opportunistic infections in humans with immunodeficiency including those with cystic fibrosis, HIV/AIDS, and pre-existing respiratory pathology (Hawkins et al., 1986; Prince et al., 1989). The bacterium is capable of establishing infection in both the intestinal and the respiratory epithelia where it ultimately invades and infects sub-mucosal macrophages. There, the bacterium establishes an intracellular niche where it can disseminate via lymph nodes and the blood (Petrofsky and Bermudez, 2005). Current efforts are being taken to understand the mechanisms used by MAH for the transmission and colonization of the epithelial mucosa. Identification of proteins that interact with host cells can be seen as candidates for the development of novel approaches to prevent the disease.

*C. elegans* and MAH are both abundant in the environment and there is a natural possibility of interaction between the two organisms. In this study, the nematode *C. elegans* is characterized for the first time as a model organism to investigate MAH infection and virulence. We describe here how *C. elegans* feeds on MAH without significant consequences on its health or lifespan. Our results also demonstrate that MAH is able to colonize the intestinal tract of the worm in a stable non-transient manner, and that colonization of the gut results from a close association between the pathogen and the apical membrane and microvilli of the intestinal epithelium. Establishing *C. elegans* as a model system for MAH infection allows for the further characterization of the pathogenic mechanisms employed by MAH, and for increased progress towards therapeutic and prevention strategies.

## RESULTS

### *C. elegans* are able to feed on MAH

**Fig. 1.**
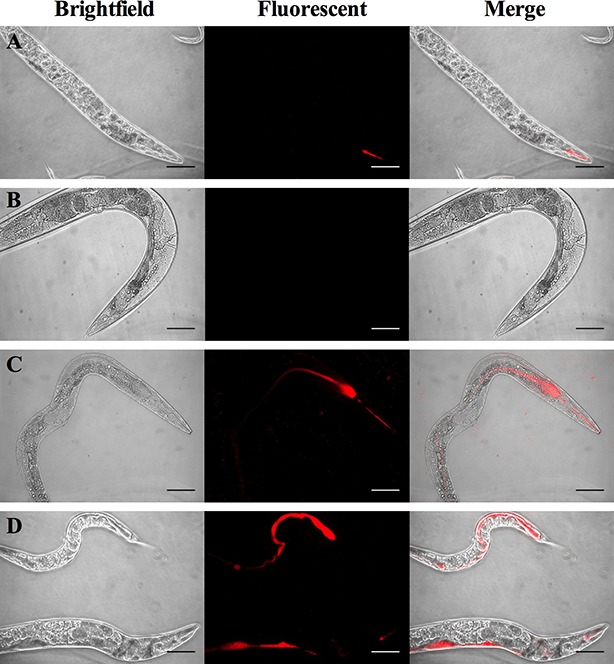
***C. elegans* feed on MAH.** *C. elegans* were seeded onto NGM plates supplemented with FUdR (400 μM) and seeded with live MAH (C,D) or heat-killed MAH (B) each containing a fluorescent red marker. Worms were fed on *E. coli* OP50 with the fluorescent red marker for 24 h as a processing and image control (A). Worms were allowed to feed for 1 (A-C) or 5 days (D) at which time worms were collected, washed, and mounted on glass slides for microscopic observation. Images are representative of 20 worms visualized per experiment and independently repeated 5 times. All images are shown at 400× magnification; scale bars are 50 μm.

It is well documented that *C. elegans* can be used as an infection model for a variety of pathogens (Couillault and Ewbank, 2002; Balla and Troemel, 2013). Previous work has determined that nematodes can be cultivated on fast growing strains of *Mycobacterium fortuitum* and *Mycobacterium marinum* (Couillault and Ewbank, 2002). Our investigation first examined whether nematodes would feed on the slow growing species MAH. NGM plates were seeded with 10^8^ MAH strain 104 containing the plasmid pJDC60-tdTomato which contains a tomato red fluorescent protein under a constitutive mycobacterial L5 promotor for identification of MAH within the intestinal tract of the nematode (MAH-td104). Synchronized worms in the L4 growth stage were seeded onto MAH-td104 containing plates and allowed to feed for 1, 3, and 5 days. Visualization of worms at each timepoint demonstrated that *C. elegans* fed on MAH-td104 in a time-dependent manner with pharynx-, grinder-, and intestinal-localized MAH-td104 increasing in intensity over time (data not shown). During the 5 days incubation, worms were able to readily feed, mate, and produce apparently healthy progeny (observation; data not shown). In order to determine if the variety of life stages played a role in uptake and feeding on MAH-td104, 5-fluoro-2′-deoxyuridine (FUdR) was added to MAH seeded NGM plates. The addition of FUdR inhibits DNA synthesis, thus preventing progeny to be produced and allows for the maintenance of a synchronous population during experiments and prevents the over population of a plate during longer timepoints. Synchronized cultures at the L4 stage of growth seeded onto NGM-FUdR plates demonstrated similar feeding trends on MAH-td104 during an identical feeding time course (Fig. 1).

Previous work demonstrated that fluorescence does not indicate viability, as GFP can still be detected within the intestine after bacteria are killed and digested for nutrients (Hsiao et al., 2013). To identify whether intestinal-localized MAH-td104 was viable, worms fed for 5 days were collected, treated with levamisole to maintain the state of bacterial uptake by preventing pharyngeal uptake or expulsion by the worms, and were treated with amikacin to remove residual extracellular MAH from the feeding assays. Quantification of worm lysates indicated that the intracellular MAH visualized after a 5-day feeding was viable as greater than 10^5^ MAH were isolated from each culture of MAH-fed nematodes. From these data, we determined that *C. elegans* would feed on MAH if it is the sole source of bacteria during growth, and that the bacteria reside within the digestive tract after ingestion.

### *C. elegans* survival upon MAH-td104 feeding

**Fig. 2.**
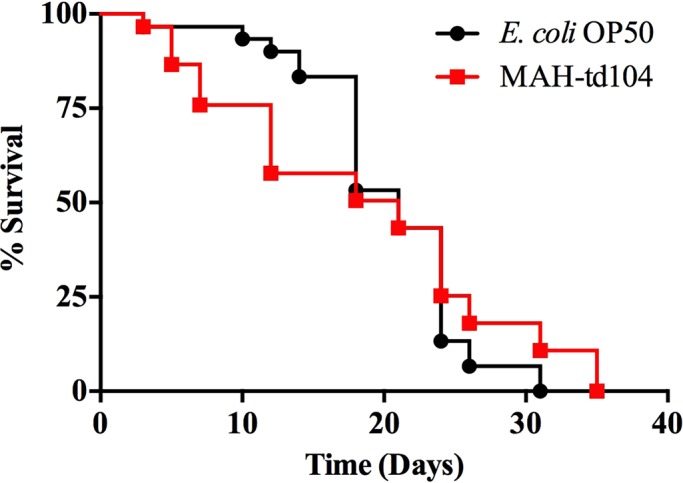
**MAH-td104 does not affect median or total lifespan of *C. elegans.*** Worms were picked and incubated on NGM agar to remove external bacteria for 3 h. Thirty worms were individually placed onto NGM-FUdR (400 µM) plates and seeded with either 10^8^ *E. coli* strain OP50 or MAH-td104. Worms were scored every 2 days for survival and worms that ruptured or crawled up the sides of the plate were censored and removed from the study. Kaplan–Meier statistics were used to construct and analyze growth characteristics. Data represents survival from one experiment and is representative of 2 independently completed experiments.

While *C. elegans* can feed on a variety of bacteria during the experimental studies, such pathogens, notably *Salmonella* and *Pseudomonas,* are rapidly lethal (Irazoqui et al., 2010). It is unknown whether the high fatty acid, lipid-rich composition of mycobacteria has an effect on the longevity of worms in culture. Feeding on MAH can be visualized for 5 days, as indicated by fluorescent microscopy (Fig. 1D); however, that timeframe may not be adequate to establish whether MAH is toxic or lethal to the nematodes. To determine lethality of MAH on *C. elegans*, a survival curve was conducted in order to compare the lifespan of *C. elegans* fed on standard *E. coli* strain OP50 cultures to the lifespan of those fed on MAH-td104. Equal numbers of synchronized worms were picked and fed on *E. coli* or MAH in the presence of FUdR for 35 days. Kaplan–Meier survival analysis indicated that feeding on MAH-td104 had no observable effect on worm lifespan as median survival of both the MAH-fed animals and OP50-fed animals is 18 days, after the start of feeding (Fig. 2). Furthermore, there is no significant difference between the total lifespan of OP50-fed worms (31 days) and that of MAH-fed worms (35 days).

### MAH colonization of the *C. elegans* intestine

**Fig. 3.**
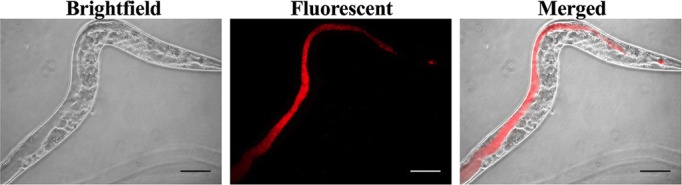
**MAH colonization persists after pulse-chase with *E. coli* strain OP50.** *C. elegans* were placed onto NGM-FUdR (400 μg/ml) plates and seeded with 10^8^ MAH-td104 and allowed to feed for 5 days. Worms were collected and moved to a new plate to remove extracellular bacteria. Worms were then transferred to a plate seeded with *E. coli* strain OP50, and allowed to feed for 24 h. Nematodes were collected and mounted onto glass slides for microscopic observation. Images are representative of 20 worms visualized per experiment and independently repeated 3 times. All images are shown at 400× magnification; scale bars are 50 μm.

*C. elegans* is capable of ingesting a wide variety of environmental bacteria for nutrients. As the nematodes can feed on MAH, the colonization appears to remain within the intestinal tract at 5 days post-infection. We analyzed whether the presence of MAH within the intestine was transient, or if it was a longer-lived, more permanent state of colonization. To answer this question, we used pulse-chase analysis during feeding. Nematodes were fed MAH-td104 for 5 days at which point they were selected and placed onto a clean NGM plate for 2 h to remove any MAH that was on the outside of the worm bodies. Worms were then placed onto a plate containing *E. coli* strain OP50 and fluorescent microscopy indicated that after 24 h of pulse-chase feeding on *E. coli*, red MAH-td104 was still readily visible within the intestinal lumen of the worms (Fig. 3).

### Visualization of the MAH-colonized *C. elegans* intestine

**Fig. 4.**
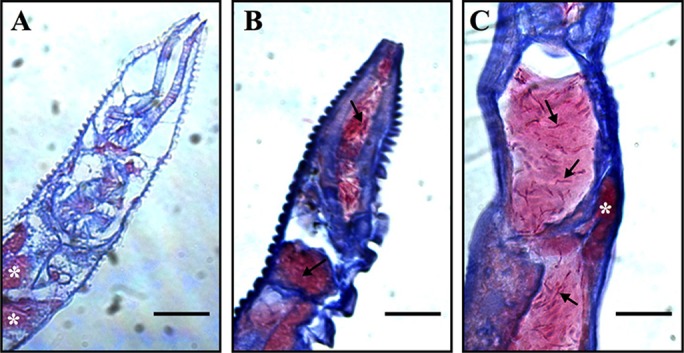
**MAH colonize lumen of *C. elegans* intestinal tract.** Worms were seeded onto NGM-FUdR (400 µM) plates for 5 days and samples were fixed and set into agarose blocks. Paraffin embedded samples were sectioned and acid-fast stained. Starved worms were seeded onto plates in the absence of bacteria (A) or onto plates containing MAH (B,C). Acid-fast positive bacilli within the intestinal space are indicated by arrows. Non-specific staining of fat deposits by carbol-fuchsin is indicated by asterisks. Images are representative of 10 worms sectioned and analyzed per treatment. All images are shown at 630× magnification; scale bars are 20 μm.

Fluorescent microscopy analysis of MAH-fed worms indicates that the bacteria are capable of colonizing the intestine of the worms during infection. As MAH is an intracellular pathogen known for its ability to invade and survive within host cells we wanted to determine whether the red fluorescent MAH seen in our photos were in the lumen of the intestine or if the bacteria were able to invade the intestinal epithelium and cause an intracellular infection within the intestinal tract. To analyze the cross-sections of MAH-fed animals, worms were seeded onto plates and fed bacteria as described above. Worms were picked at 5-days post-infection and fixed for histology. As a control, worms were seeded onto plates in the absence of any bacteria for the same incubation time and processed as described. Acid-fast stained cross-sections indicate that MAH was capable of colonizing the lumen of the intestine at high levels during feeding and infection (Fig. 4B and C), while starved worms indicate an absence of pink acid-fast bacilli, or any bacilli within the lumen of the intestine (Fig. 4A).

**Fig. 5.**
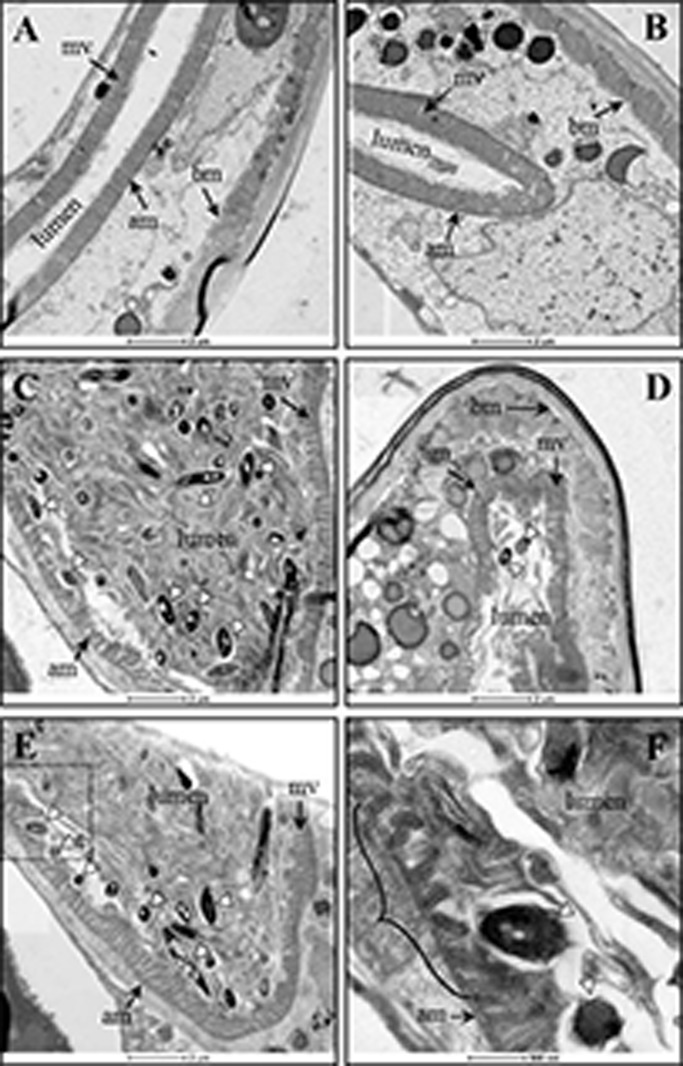
**Transmission electron microscopy of MAH-colonized *C. elegans.*** Worms were seeded onto NGM-FUdR (400 µM) plates for 5 days and samples were fixed, processed, and visualized by transmission electron microscopy on an FEI Titan 80-200 microscope. Starved worms were seeded onto plates in the absence of bacteria (A,B) or onto plates seeded with 10^8^ MAH (C-F). F illustrates a magnified view taken from E (dotted line) and shows disruption on the microvilli (bracket). Key: arrowheads, MAH; lumen, luminal space; mv, microvilli; am, apical epithelial membrane; bm, basal epithelial membrane. A-E, scale bar is 2 µm; F, scale bar is 500 nm.

In order to visualize if MAH interacts with the intestinal epithelium of *C. elegans* following feeding, MAH-fed and starved worms were prepared and visualized using transmission electron microscopy. As a healthy and uninfected control, starved worms demonstrate an absence of bacteria within the lumen which appears compact in size (Fig. 5A and B). An undisturbed intestinal tract can be clearly seen in the control worms as intact and tightly clustered microvilli line the apical membrane of the intestinal epithelial cells. Alternatively, MAH-fed worms present with a grossly distended lumen that is filled with colonizing MAH bacteria (Fig. 5C-F). MAH can be seen both in the luminal space, as well as in direct contact with the microvilli located on the apical membrane of the intestinal epithelium. Both at the site of contact, as well as in the vicinity of these sites, it can be appreciated that the neatly layered, tightly clustered microvilli seen in the control sections are shortened, appear damaged, and in a much looser association in the presence of MAH colonization (Fig. 5C-F).

### Binding and colonization of MAH glycopeptidolipid mutant ΔGPL/4B2

**Fig. 6.**
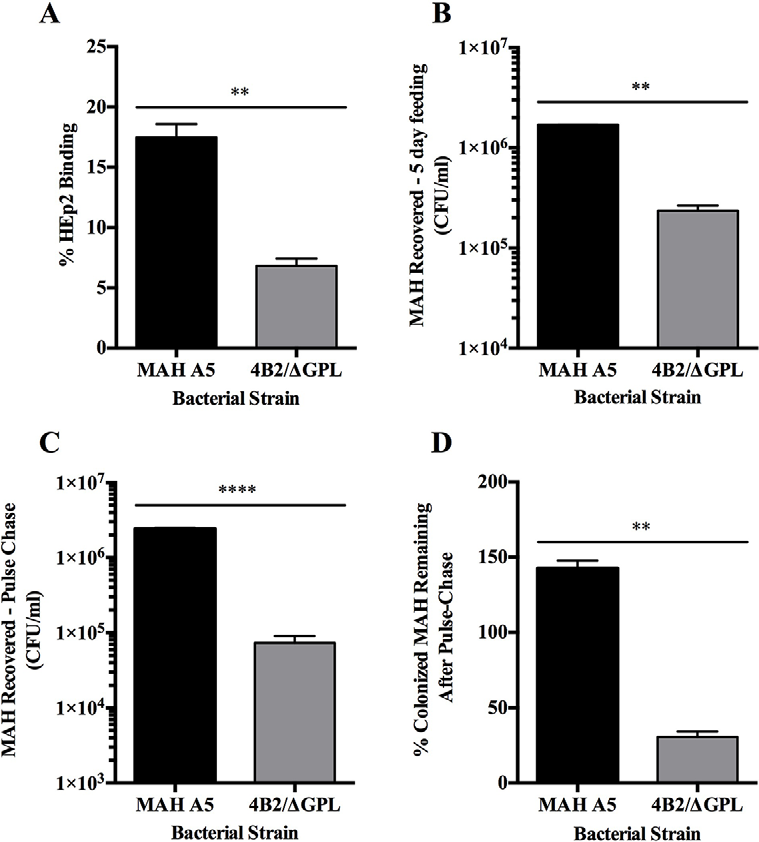
**Binding of HEp-2 cells and colonization of *C. elegans* by MAH ΔGPL/4B2 mutant.** (A) The MAH 4B2/ΔGPL mutant and the parental strain MAH A5 were used for HEp-2 binding assays. HEp-2 cells were infected at an MOI of 10:1 with each strain and binding was allowed to progress for 1 h at 4°C. Wells were lysed and quantified for percent of bound bacteria to the surface of epithelial cells during assay. Equivalent numbers of *C. elegans* were seeded onto NGM-FUdR (400 µM) plates containing 10^8^ of each MAH strain and allowed to feed at 25°C for 5 days. Worms were collected, washed with levamisole (25 mM), treated with amikacin (200 µg/ml), and lysed for quantification of intestinal bacteria. (B) Worms were homogenized immediately after MAH feeding to determine intestinal binding ability after feeding. (C) Analysis for colonization using pulse-chase assays were conducted by transferring worms to NGM-FUdR plates seeded with *E. coli* strain OP50 for 24 h prior to homogenization and quantification. (D) The percent of each MAH strain remaining from the initial 5 days feeding after the pulse-chase was conducted was calculated by (MAH recovered−5 day feeding/MAH recovered−Pulse-Chase)×100. Data represent the mean±s.e.m. of 2 independent experiments each performed in triplicate (**P<0.01, ****P<0.0001 as determined by Student's *t*-test).

We next aimed to determine if the *C. elegans* model could identify MAH mutants and lead to the understanding of bacterial components responsible for the colonization of the intestinal epithelium within the nematode. The previously described MAH 4B2/ΔGPL mutant (Yamazaki et al., 2006b) was analyzed for binding to HEp-2 epithelial cells during a 1 h infection (Fig. 6A). Compared to the parental MAH A5 strain the MAH 4B2/ΔGPL mutant is deficient in its ability to bind to human HEp-2 cells during infection. Worms were allowed to feed on the wild-type and mutant MAH strains for 5 days and intestinal binding was quantified to assess the ability of worms to feed on the bacteria and the intestinal colonization of each bacterial strain at that timepoint (Fig. 6B). The MAH 4B2/ΔGPL mutant is found at significantly lower levels than the wild-type MAH strain within the intestinal tract of the worms. After 5 days of feeding on each bacterial strain, nematodes underwent pulse-chase analysis with a 24-h feeding on *E. coli* strain OP50 prior to intestinal MAH quantification (Fig. 6C). The ability of the MAH 4B2/ΔGPL mutant to colonize the *C. elegans* intestinal epithelium is lower compared to the wild-type MAH infection as indicated by a significant decrease in the amount of bacteria localized in the intestine after pulse-chase analysis. Furthermore, when the initial amount of bacteria present at 5 days of feeding, and the remaining bacteria present after pulse-chase are compared, it is evident that that MAH 4B2/ΔGPL mutant has a significant deficiency in its ability to maintain colonization within the intestinal lumen of the nematode compared to wild-type MAH A5 (Fig. 6D).

## DISCUSSION

*C. elegans* has been illustrated as an extremely useful model system used to study a variety of research questions from genetics and cell biology aspects, to pathogenesis and host-microbe interactions. The transparent nature of the nematode and simple body structure provides a simplified *in vivo* model to mimic physiological and pathogenic mechanisms that occur during infection. The manipulation and use of *C. elegans* is simple, inexpensive, and there are a variety of tools available for studying the processes occurring within the host. *C. elegans* provides an intestinal physiology that is relevant for the study of MAH infection. The bacterium is known to invade the enterocytes of the intestinal epithelium, rather than the Peyer's Patches and M-cells that are used by other mycobacteria (Sangari et al., 2001). Likewise, the relatively similar physiology and epithelial make-up of the intestinal epithelium of the enterocytes provides a high degree of similarity and hypothesized interaction between bacterial pathogens such as mycobacterium and the host during infection.

In this study, we show that *C. elegans* can serve as a useful tool and model system to understand the pathogenesis of MAH, specifically its interaction and infection of the intestinal epithelium. Nematodes naturally feed on environmental bacteria for nutrients and we show that the ability of populations *C. elegans* to feed on MAH is no exception. Unlike the feeding on other pathogens, such as *Pseudomonas* and *Salmonella,* the ingestion of MAH is not rapidly toxic. These observations suggest that MAH does not exude rapidly toxic components within the intestinal tract of the worm during infection resulting in a longer-lived infection within the *C. elegans* host. In fact, this finding should not represent a surprise, since both MAH and *C. elegans* co-inhabit the same environmental niche.

It is described that *M. avium* first colonizes the host intestinal epithelium prior to being observed as systemic bacterial infection (Roth et al., 1985). Our histopathological images and the pulse-chase data demonstrate that MAH are found within the lumen of the intestinal tract over the 5-day infection, quickly increasing in number and colonizing in a stable, non-transient manner. These data are supported by TEM images of MAH-infected worms, which indicate that MAH establish a direct interaction during colonization with the intestinal epithelium. This interaction results in damage to the villi on the epithelial cells, which has been observed during mouse infection (Kim et al., 1998). As the current experimental infection only progressed until 5-days post-feeding (obtained images), it is possible that longer infection of *C. elegans* with MAH would cause progression of the intestinal pathology. These observations suggest that MAH is able to colonize the intestinal epithelium to very likely, establish a more long-term and chronic infection within the host.

Past experimental data indicates that the MAH ΔGPL/4B2 mutant, which expresses a decreased ability to form biofilm on surfaces (Yamazaki et al., 2006a), and an impaired capacity to bind to human epithelial HEp-2 cells, also shows a decreased interaction with the intestinal tract of *C. elegans*, with deficiency in binding to and colonization of the epithelium*.* The model, therefore, may be quite useful to screen and identify mutants with impaired ability to colonize the host mucosa surface. In fact, many studies have identified bacterial molecules associated with invasion of the host mucosal by bacteria, but not much is known about the ability of MAH to anchor to structures on the epithelium (Dam et al., 2006; Harriff et al., 2009). Ultimately, we aim to understand the bacterial mechanisms and factors that are responsible for MAH colonization and infection of the host. The identification of such factors used to initiate the disease could be useful in developing therapeutic approaches to prevent the infection from establishing or to treat infections that are in the early stages of colonization.

## METHODS AND MATERIALS

### Nematode propagation

*Caenorhabditis elegans* (*C. elegans*) strain N2 was kindly provided by the laboratory of Dr Dee Denver at Oregon State University. Nematodes were maintained in monoxenic cultures with the addition of *Escherichia coli* strain OP50 and propagated on nematode growth medium (NGM) agar plates at 25°C as previously described (Brenner, 1974).

### Bacterial culture

*E. coli* strain OP50 was grown in Luria-Bertani (Roth et al., 1985) broth overnight prior to inoculation of NGM plates. *Mycobacterium avium* subspecies *hominissuis* (MAH) strain 104 and strain A5 was grown on Middlebrook 7H10 agar supplemented with 10% w/v oleic acid-albumin-dextrose-catalase (OADC; Hardy Diagnostics; Santa Maria, CA, USA) for 10 days at 37°C. MAH A5 ΔGPL/4B2 mutant (described by Yamazaki et al., 2006a) and MAH strain 104 containing the plasmid pJDC60-tdTomato (MAH-td104) was grown on 7H10 medium described above supplemented with kanamycin sulfate (400 µg/ml). Bacterial suspensions were processed through a 23-gauge syringe, clumps allowed to settle for 10 min, and top 2 ml of suspension collected and used for assays.

### Mammalian cell culture

Human epithelial (HEp-2; CCL-23) cells were obtained from the American Type Culture Collection (ATCC; Manassas, VA, USA). Cells were grown in RPMI medium supplemented with 10% heat-inactivated fetal bovine serum (FBS; Gemini Bio-Products; West Sacramento, CA, USA) in 37°C with 5% CO_2_.

### Nematode MAH feeding assays

NGM plates were prepared as described above, or supplemented with 400 µM 5-fluoro-2′-deoxyuridine, a DNA synthesis inhibitor which allows for the synchronization of worm cultures (FUdR; Sigma-Aldrich; St. Louis, MO, USA) (Mitchell et al., 1979). Agar plates with and without FUdR were seeded with 10^8^ MAH-td104, heat-killed MAH, or the appropriate MAH mutant strain as per experimental design, and inoculated with equal volume synchronized nematode cultures at the L4 stage of growth. At 1, 3, and 5 days post-inoculation, worms were collected in M9 salt solution, pelleted, and anesthetized using 70% ethanol. Samples were spotted onto glass slides, and visualized on a DM4000B Leica microscope. Images were captured and analyzed using QCapture Pro7 software.

### Pulse-chase feeding assay

Pulse-chase analysis was modified for mycobacterial isolation and conducted as previously described (Chou et al., 2013). Synchronized worms at the L4 growth stage were collected in 1× M9 saline solution and washed twice. Individual NGM plates supplemented with FUdR (400 µM) were seeded with 10^8^ MAH as per experimental design. Each plate was seeded with equivalent number of worms and incubated at 25°C. After 5 days, worms were collected in M9 saline, washed twice, and seeded onto new NGM-FUdR plates containing a lawn of *E. coli* OP50. After 24 h of incubation, worms were collected in M9 saline supplemented with 25 mM of levamisole hydrochloride (Sigma-Aldrich) for paralysis and prevention of expulsion or uptake of bacteria during washes. Worms were collected at 225 ×***g*** for 2 min, washed twice in M9 saline solution, treated with amikacin sulfate (200 µg/ml) for 2 h at room temperature to kill all extracellular bacteria surrounding the worms, and washed twice with HBSS. For visualization, suspensions were analyzed under fluorescent microscopy. To quantify pulse-chase assays, suspensions were homogenized using a handheld motorized pestle (VWR; Radnor, PA, USA) for 1 min in 0.1% triton X-100 and deionized water, and samples were serially diluted and quantified.

### Nematode survival assay

Synchronized nematode cultures were prepared and 30 worms were picked using a platinum wire pick and transferred to NGM plates supplemented with FUdR (400 µM) which were seeded with either 10^8^ *E. coli* strain OP50 or 10^8^ MAH strain 104 and incubated at 25°C. Cultures were monitored and scored for nematode survival. Worms that did not move or respond to gentle agitation by platinum wire pick were deemed dead, and worms found on the plastic sides and lid of petri plate as well as worms that burst were censored from the data counts. Survival of populations was assessed for 30 days and analyzed using a Kaplan–Meier survival analysis.

### Histology and transmission electron microscopy

#### Specimen preparation

Synchronized nematodes at the L4 growth stage were picked and seeded onto NGM agar plates supplemented with FUdR (400 µM) for 24 h in the absence of any seeded bacteria to remove any extracellular or residual *E. coli* from the worm cultures. Worms were then picked and seeded onto plates containing either 10^8^ MAH-td104 for experimentally fed samples or containing no bacteria for starved control samples and allowed to incubate for 5 days at 25°C.

#### Histology

Worms were collected in M9 saline solution and washed twice at 225 ×***g*** for 2 min to remove any extracellular bacteria in suspension. Nematodes were fixed in 10% buffered formalin for 5 min at room temperature, washed in M9 solution, suspended in 1% low-melt agarose. Agarose-encased worms were embedded in resin, and sections mounted onto glass slides by the Veterinary Diagnostic Lab at Oregon State University. Specimens were acid-fast stained and visualized.

#### Transmission electron microscopy

Worms were collected in M9 solution, washed twice, and pellet was suspended in fixative buffer of 2.5% glutaraldehyde, 1% paraformaldehyde, and 0.1 M sodium cacodylate. Worms were cut in half and incubated in fixative buffer overnight at 4°C. Specimen sections were stained, dehydrated, and infiltrated for TEM visualization by the Electron Microscopy Facility at Oregon State University as previously described (Hall, 1995).

### HEp-2 binding assay

Bacterial suspensions were prepared in Hank's Balanced Salt Solution (HBSS; Corning; Corning, NY, USA) and HEp-2 cells were infected at an MOI of 10:1. Infections were synchronized at 225 ×***g*** for 5 min at 4°C. Plates were incubated for 1 h at 4°C, washed 3 times with PBS, and monolayers lysed for 15 min in 0.1% Triton X-100 in water. Lysates were serially diluted and quantified using CFU counts.

### Statistical analysis and data interpretation

Results are reported as the mean of at least 2 independent experiments±standard error (s.e.m). For binding and pulse-chase assays statistical comparisons between experimental groups and control groups were determined using the Student's *t-*test with *P*<0.05 denoting statistical significance. Survival curve data was analyzed using Kaplan–Meier Survival Analysis. GraphPad Prism version 6.0 software was used for the construction of graphs, data interpretation, and all statistical analyses.
